# Tetra­quabis(5-fluoro­saccharinato)nickel(II)

**DOI:** 10.1107/S1600536809007053

**Published:** 2009-03-06

**Authors:** Larnelle Peterson, Jennifer Kelley, LeRoy Peterson, Mark D. Smith, Hans-Conrad zur Loye

**Affiliations:** a Chemistry Department, Francis Marion University, Florence, South Carolina 29501, USA; b Department of Chemistry and Biochemistry, University of South Carolina, Columbia, South Carolina 29208, USA

## Abstract

In the centrosymmetric title complex, [Ni(C_7_H_3_FNO_3_S)_2_(H_2_O)_4_], the Ni^II^ atom exhibits a slightly distorted *trans*-NiN_2_O_4_ octa­hedral coordination. The nitro­gen donors are provided by two 5-fluoro­saccharinate ligands and the oxygen donors are provided by four water mol­ecules. The crystal structure features O—H⋯O and bifurcated O—H⋯(F,O) hydrogen bonds, the latter involving the F atom of the 5-fluoro­saccharinate ligand.

## Related literature

For a related structure; see: Haider *et al.* (1983 ▶). For background, see: Falvello *et al.* (2001 ▶); Khalil *et al.* (2005 ▶); Plenio (1997 ▶).
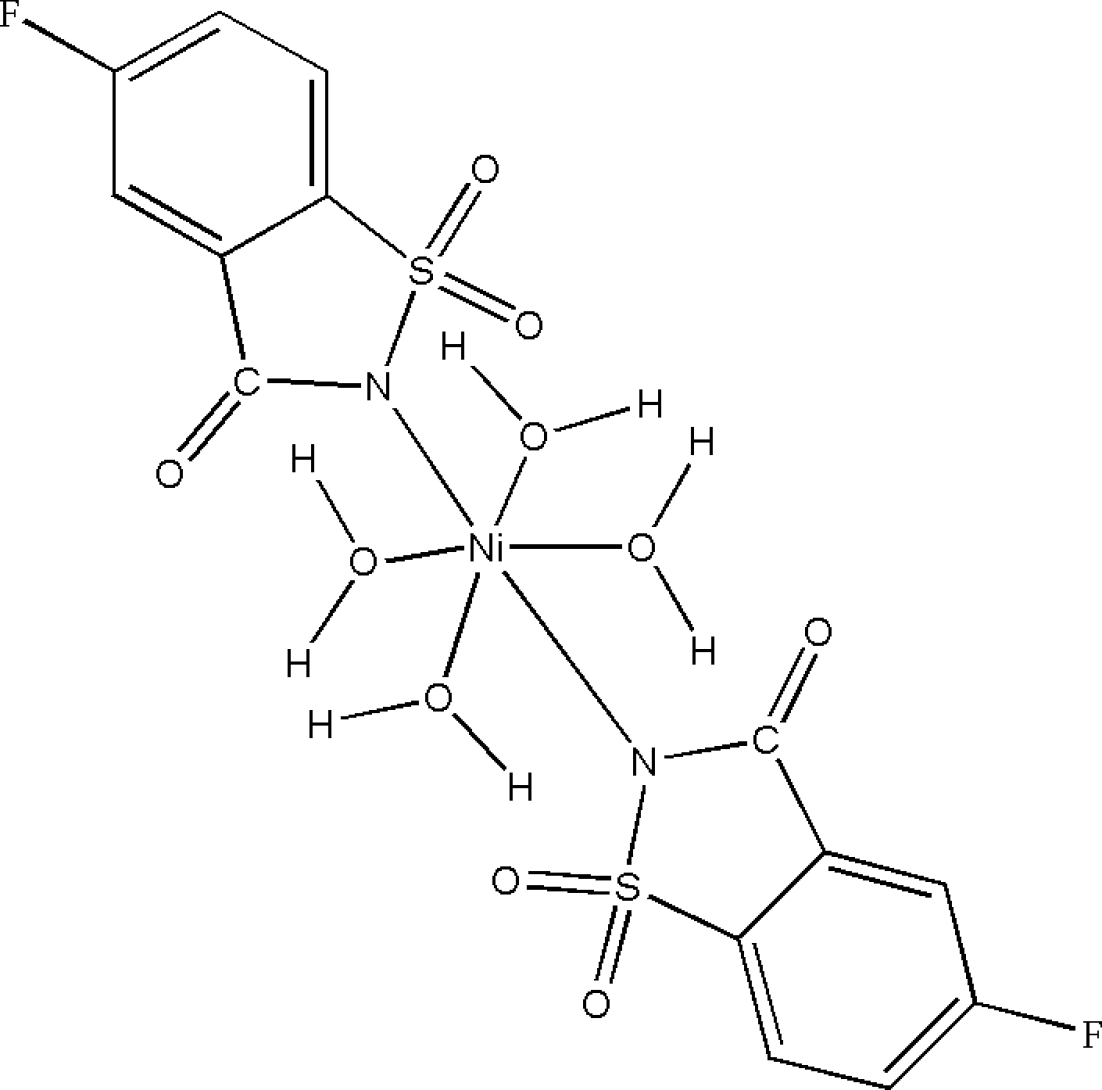


## Experimental

### 

#### Crystal data

[Ni(C_7_H_3_FNO_3_S)_2_(H_2_O)_4_]
*M* *_r_* = 531.10
Triclinic, 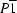

*a* = 6.9649 (3) Å
*b* = 8.0484 (3) Å
*c* = 9.5877 (4) Å
α = 101.780 (1)°
β = 105.983 (1)°
γ = 110.973 (1)°
*V* = 454.18 (3) Å^3^
*Z* = 1
Mo *K*α radiation
μ = 1.38 mm^−1^
*T* = 150 K
0.22 × 0.18 × 0.08 mm

#### Data collection

Bruker SMART APEX CCD diffractometer
Absorption correction: multi-scan (*SADABS*; Bruker, 2003 ▶) *T*_min_ = 0.887, *T*_max_ = 1.000 (expected range = 0.794–0.895)
6861 measured reflections
1858 independent reflections
1769 reflections with *I* > 2σ(*I*)
*R*_int_ = 0.022

#### Refinement

*R*[*F* ^2^ > 2σ(*F* ^2^)] = 0.024
*wR*(*F* ^2^) = 0.065
*S* = 1.06
1858 reflections
161 parameters
Only H-atom displacement parameters refined
Δρ_max_ = 0.38 e Å^−3^
Δρ_min_ = −0.37 e Å^−3^

### 

Data collection: *SMART* (Bruker, 2003 ▶); cell refinement: *SAINT-Plus* (Bruker, 2003 ▶); data reduction: *SAINT-Plus*; program(s) used to solve structure: *SHELXS97* (Sheldrick, 2008 ▶); program(s) used to refine structure: *SHELXL97* (Sheldrick, 2008 ▶); molecular graphics: *DIAMOND* (Brandenburg, 2005 ▶); software used to prepare material for publication: *SHELXTL* (Sheldrick, 2008 ▶).

## Supplementary Material

Crystal structure: contains datablocks I, global. DOI: 10.1107/S1600536809007053/hb2907sup1.cif

Structure factors: contains datablocks I. DOI: 10.1107/S1600536809007053/hb2907Isup2.hkl

Additional supplementary materials:  crystallographic information; 3D view; checkCIF report

## Figures and Tables

**Table 1:** Selected bond lengths (Å)

Ni1—O5	2.0440 (13)
Ni1—N1	2.0856 (14)
Ni1—O4	2.1084 (13)

**Table 2:** Hydrogen-bond geometry (Å, °)

*D*—H⋯*A*	*D*—H	H⋯*A*	*D*⋯*A*	*D*—H⋯*A*
O4—H4*A*⋯O2	0.81 (3)	2.51 (3)	3.1436 (19)	137 (3)
O4—H4*A*⋯F1^i^	0.81 (3)	2.54 (3)	3.0910 (19)	127 (3)
O4—H4*B*⋯O3^ii^	0.78 (3)	2.17 (3)	2.8985 (18)	158 (3)
O5—H5*A*⋯O3	0.80 (3)	2.10 (3)	2.8346 (18)	155 (3)
O5—H5*A*⋯F1^iii^	0.80 (3)	2.59 (3)	3.1050 (17)	124 (2)
O5—H5*B*⋯O1^iv^	0.81 (3)	2.13 (3)	2.793 (2)	139 (2)
O5—H5*B*⋯O2^ii^	0.81 (3)	2.44 (3)	2.9541 (18)	122 (2)

Symmetry codes: (i) 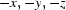; (ii) 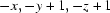; (iii) 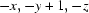; (iv) 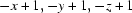.

**Fig. 1.**
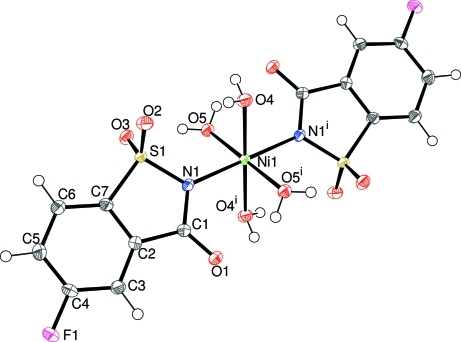
The coordination environment of Ni(II) in (I), with the atom-labeling scheme. The H atoms of 5-fsacch are omitted for clarity. Displacement ellipsoids for nonhydrogen atoms are drawn at the 50% probability level. Hydrogen bonds are represented by dashed lines.

**Fig. 2.**
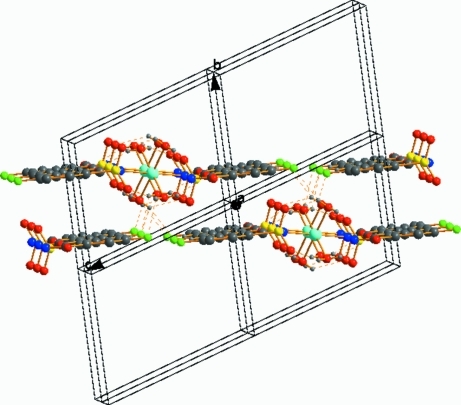
View of the crystal packing in (I). All H atoms except for those of water are omitted for clarity. Hydrogen bonds are represented by dashed lines.

## supplementary crystallographic information

### Comment

The study of metal saccharinate complexes has been of current interest with respect to their incorporation into novel coordination polymers (Falvello *et al.*, 2001). As a continuation of our own efforts in this area (Khalil *et al.*, 2005), we have deemed it worthwhile to explore the solid state structures of metal organic compounds containing fluorinated saccharinates. The choice of fluorinated saccharinates stems from the novel types of interactions in which carbon bound fluorine may participate (Plenio, 1997). Our initial studies have led to the preparation of the title nickel complex (I) that contains 5-fluorosaccharinate (5-fsacch) as an anionic ligand.

The crystal structure of (I) consists of monomeric Ni(5-fsacch)_2_(H_2_O)_4_ molecular units, as shown in Figure 1. The Ni^II^ atom, which lies on an inversion center, is octahedrally coordinated by a pair of *trans* N atoms from two equivalent 5-fsacch ligands, and by four O atoms from two pairs that contain equivalent water molecules (Table 1).

The average Ni—N and Ni—O bond distances in (I) are 2.086 Å (1) and 2.076 (2) Å, respectively. By comparison to a similar structure, in (I) the average Ni—N distance is shorter whereas the average Ni—O distance is longer than their corresponding values in the previously reported nickel saccharinate complex, namely Ni(sacch)_2_(H_2_O)_4_.2(H_2_O) (II) (sacch = saccharinate) (Haider *et al.*, 1983). In (II) the average Ni—N distance is 2.154 (1) Å, while the average Ni—O distance is 2.069 (2) Å. All angles in (I) are normal and are comparable to their corresponding values in (II).

The crystal structure in (I) features extensive hydrogen bonding (Table 2) in which both the carbonyl and sulfonyl O atoms of 5-fsacch, as well its carbon bound fluorine, act as hydrogen bond acceptors for the water H atoms, as shown in Fig. 2. This hydrogen bonding scheme is different from that of (II) for two major reasons. First, there is the presence of the previously mentioned C—F···H hydrogen bonding in (I) that is obviously absent in (II). Second, in (II) there exists hydrogen bonding involving lattice water molecules, which because of their absence in (I) precludes such interactions.

### Experimental

All chemicals and solvents were purchased from commercial sources and used without further purification. The synthesis of sodium 5-fluorosaccharinate will be described elsewhere. A 10 ml solution of sodium 5-fluorosaccharinate (0.10 mmol) was added dropwise to a 10.0 ml solution of nickel(II) chloride tetrahydrate (0.050 mmol). Light blue, block-like crystals of (I) were formed in about three weeks by slow evaporation after the solution volume was reduced to 5.0 ml under ambient conditions.

### Refinement

Hydrogen atoms bonded to carbon were placed in geometrically idealized positions and included as riding atoms with refined isotropic displacement parameters. The water H atoms were located in difference maps and refined freely.

### Crystal data

**Table:**

[Ni(C_7_H_3_FNO_3_S)_2_(H_2_O)_4_]	*Z* = 1
*M**_r_* = 531.10	*F*(000) = 270
Triclinic, *P*1	*D*_x_ = 1.942 Mg m^−^^3^
Hall symbol: -P 1	Mo *K*α radiation, λ = 0.71073 Å
*a* = 6.9649 (3) Å	Cell parameters from 4507 reflections
*b* = 8.0484 (3) Å	θ = 2.4–26.4°
*c* = 9.5877 (4) Å	µ = 1.38 mm^−^^1^
α = 101.780 (1)°	*T* = 150 K
β = 105.983 (1)°	Block, light blue
γ = 110.973 (1)°	0.22 × 0.18 × 0.08 mm
*V* = 454.18 (3) Å^3^	

### Data collection

**Table:**

Bruker SMART APEX CCD diffractometer	1858 independent reflections
Radiation source: fine-focus sealed tube	1769 reflections with *I* > 2σ(*I*)
graphite	*R*_int_ = 0.022
ω scans	θ_max_ = 26.4°, θ_min_ = 2.4°
Absorption correction: multi-scan (*SADABS*; Bruker, 2003)	*h* = −8→8
*T*_min_ = 0.887, *T*_max_ = 1.000	*k* = −10→10
6861 measured reflections	*l* = −11→11

### Refinement

**Table:**

Refinement on *F*^2^	Primary atom site location: structure-invariant direct methods
Least-squares matrix: full	Secondary atom site location: difference Fourier map
*R*[*F*^2^ > 2σ(*F*^2^)] = 0.024	Hydrogen site location: mixed
*wR*(*F*^2^) = 0.065	Only H-atom displacement parameters refined
*S* = 1.06	*w* = 1/[σ^2^(*F*_o_^2^) + (0.0376*P*)^2^ + 0.2342*P*] where *P* = (*F*_o_^2^ + 2*F*_c_^2^)/3
1858 reflections	(Δ/σ)_max_ < 0.001
161 parameters	Δρ_max_ = 0.38 e Å^−^^3^
0 restraints	Δρ_min_ = −0.37 e Å^−^^3^

### Special details

**Table:**

Geometry. All e.s.d.'s (except the e.s.d. in the dihedral angle between two l.s. planes) are estimated using the full covariance matrix. The cell e.s.d.'s are taken into account individually in the estimation of e.s.d.'s in distances, angles and torsion angles; correlations between e.s.d.'s in cell parameters are only used when they are defined by crystal symmetry. An approximate (isotropic) treatment of cell e.s.d.'s is used for estimating e.s.d.'s involving l.s. planes.
Refinement. Refinement of *F*^2^ against ALL reflections. The weighted *R*-factor *wR* and goodness of fit *S* are based on *F*^2^, conventional *R*-factors *R* are based on *F*, with *F* set to zero for negative *F*^2^. The threshold expression of *F*^2^ > σ(*F*^2^) is used only for calculating *R*-factors(gt) *etc*. and is not relevant to the choice of reflections for refinement. *R*-factors based on *F*^2^ are statistically about twice as large as those based on *F*, and *R*- factors based on ALL data will be even larger.

### Fractional atomic coordinates and isotropic or equivalent isotropic displacement parameters (Å^2^)

**Table:**

	*x*	*y*	*z*	*U*_iso_*/*U*_eq_	
Ni1	0.5000	0.5000	0.5000	0.01450 (11)	
S1	−0.01342 (6)	0.32955 (6)	0.24583 (5)	0.01495 (12)	
C1	0.2752 (3)	0.2997 (2)	0.1523 (2)	0.0176 (4)	
C2	0.0749 (3)	0.2377 (2)	0.0098 (2)	0.0164 (3)	
C3	0.0542 (3)	0.1736 (2)	−0.1416 (2)	0.0179 (3)	
H3	0.1724	0.1621	−0.1670	0.020 (5)*	
C4	−0.1482 (3)	0.1274 (2)	−0.2531 (2)	0.0179 (3)	
C5	−0.3263 (3)	0.1414 (3)	−0.2223 (2)	0.0217 (4)	
H5	−0.4613	0.1090	−0.3041	0.036 (6)*	
C6	−0.3043 (3)	0.2036 (3)	−0.0695 (2)	0.0209 (4)	
H6	−0.4232	0.2132	−0.0438	0.026 (6)*	
C7	−0.1013 (3)	0.2507 (2)	0.04337 (19)	0.0169 (3)	
F1	−0.17280 (17)	0.06728 (16)	−0.40248 (12)	0.0230 (2)	
N1	0.2420 (2)	0.3605 (2)	0.28345 (17)	0.0172 (3)	
O1	0.4490 (2)	0.2954 (2)	0.15047 (15)	0.0258 (3)	
O2	−0.1302 (2)	0.18308 (18)	0.29833 (15)	0.0210 (3)	
O3	−0.01877 (19)	0.50819 (17)	0.30498 (14)	0.0193 (3)	
O4	0.2988 (2)	0.3398 (2)	0.59836 (16)	0.0197 (3)	
H4A	0.190 (5)	0.249 (4)	0.535 (4)	0.048 (8)*	
H4B	0.254 (5)	0.396 (4)	0.646 (3)	0.043 (8)*	
O5	0.4047 (2)	0.71025 (18)	0.54925 (17)	0.0187 (3)	
H5A	0.284 (5)	0.685 (4)	0.490 (3)	0.046 (8)*	
H5B	0.412 (4)	0.742 (4)	0.637 (3)	0.039 (7)*	

### Atomic displacement parameters (Å^2^)

**Table:**

	*U*^11^	*U*^22^	*U*^33^	*U*^12^	*U*^13^	*U*^23^
Ni1	0.01254 (16)	0.01878 (18)	0.01260 (17)	0.00792 (13)	0.00498 (12)	0.00392 (12)
S1	0.0128 (2)	0.0187 (2)	0.0134 (2)	0.00758 (17)	0.00549 (16)	0.00357 (17)
C1	0.0179 (8)	0.0214 (9)	0.0152 (8)	0.0101 (7)	0.0070 (7)	0.0056 (7)
C2	0.0156 (8)	0.0175 (8)	0.0163 (9)	0.0077 (7)	0.0058 (7)	0.0058 (7)
C3	0.0183 (8)	0.0192 (8)	0.0183 (9)	0.0092 (7)	0.0086 (7)	0.0066 (7)
C4	0.0228 (8)	0.0174 (8)	0.0126 (8)	0.0079 (7)	0.0073 (7)	0.0043 (7)
C5	0.0173 (8)	0.0242 (9)	0.0189 (9)	0.0081 (7)	0.0031 (7)	0.0052 (7)
C6	0.0168 (8)	0.0261 (9)	0.0190 (9)	0.0096 (7)	0.0070 (7)	0.0052 (7)
C7	0.0180 (8)	0.0181 (8)	0.0135 (8)	0.0079 (7)	0.0063 (7)	0.0030 (7)
F1	0.0246 (5)	0.0294 (6)	0.0125 (5)	0.0113 (5)	0.0064 (4)	0.0040 (4)
N1	0.0129 (6)	0.0241 (8)	0.0148 (7)	0.0100 (6)	0.0050 (6)	0.0040 (6)
O1	0.0188 (6)	0.0437 (8)	0.0177 (7)	0.0188 (6)	0.0073 (5)	0.0061 (6)
O2	0.0216 (6)	0.0228 (7)	0.0200 (7)	0.0090 (5)	0.0109 (5)	0.0073 (5)
O3	0.0170 (6)	0.0202 (6)	0.0196 (6)	0.0096 (5)	0.0062 (5)	0.0030 (5)
O4	0.0184 (6)	0.0213 (7)	0.0188 (7)	0.0082 (6)	0.0087 (6)	0.0044 (6)
O5	0.0170 (6)	0.0229 (7)	0.0163 (7)	0.0105 (5)	0.0060 (5)	0.0042 (5)

### Geometric parameters (Å, °)

**Table:**

Ni1—O5^i^	2.0440 (13)	C2—C3	1.385 (2)
Ni1—O5	2.0440 (13)	C3—C4	1.379 (2)
Ni1—N1	2.0856 (14)	C3—H3	0.9500
Ni1—N1^i^	2.0856 (14)	C4—F1	1.355 (2)
Ni1—O4	2.1084 (13)	C4—C5	1.388 (2)
Ni1—O4^i^	2.1084 (13)	C5—C6	1.394 (3)
S1—O2	1.4443 (13)	C5—H5	0.9500
S1—O3	1.4515 (13)	C6—C7	1.385 (2)
S1—N1	1.6277 (14)	C6—H6	0.9500
S1—C7	1.7635 (17)	O4—H4A	0.81 (3)
C1—O1	1.228 (2)	O4—H4B	0.78 (3)
C1—N1	1.366 (2)	O5—H5A	0.80 (3)
C1—C2	1.495 (2)	O5—H5B	0.81 (3)
C2—C7	1.385 (2)		
			
O5^i^—Ni1—O5	180.0	C3—C2—C1	127.30 (15)
O5^i^—Ni1—N1	87.50 (6)	C4—C3—C2	116.09 (15)
O5—Ni1—N1	92.50 (6)	C4—C3—H3	122.0
O5^i^—Ni1—N1^i^	92.50 (6)	C2—C3—H3	122.0
O5—Ni1—N1^i^	87.50 (6)	F1—C4—C3	117.60 (15)
N1—Ni1—N1^i^	180.0	F1—C4—C5	118.11 (16)
O5^i^—Ni1—O4	88.53 (5)	C3—C4—C5	124.28 (17)
O5—Ni1—O4	91.47 (5)	C4—C5—C6	119.03 (16)
N1—Ni1—O4	90.65 (6)	C4—C5—H5	120.5
N1^i^—Ni1—O4	89.35 (6)	C6—C5—H5	120.5
O5^i^—Ni1—O4^i^	91.47 (5)	C7—C6—C5	117.06 (16)
O5—Ni1—O4^i^	88.53 (5)	C7—C6—H6	121.5
N1—Ni1—O4^i^	89.35 (6)	C5—C6—H6	121.5
N1^i^—Ni1—O4^i^	90.65 (6)	C2—C7—C6	122.86 (16)
O4—Ni1—O4^i^	180.0	C2—C7—S1	107.03 (13)
O2—S1—O3	114.08 (7)	C6—C7—S1	130.10 (13)
O2—S1—N1	110.96 (8)	C1—N1—S1	111.66 (12)
O3—S1—N1	110.42 (7)	C1—N1—Ni1	122.66 (11)
O2—S1—C7	111.32 (8)	S1—N1—Ni1	125.40 (8)
O3—S1—C7	112.14 (8)	Ni1—O4—H4A	113 (2)
N1—S1—C7	96.63 (8)	Ni1—O4—H4B	113 (2)
O1—C1—N1	124.25 (16)	H4A—O4—H4B	106 (3)
O1—C1—C2	123.43 (16)	Ni1—O5—H5A	113 (2)
N1—C1—C2	112.32 (14)	Ni1—O5—H5B	113.5 (19)
C7—C2—C3	120.66 (16)	H5A—O5—H5B	110 (3)
C7—C2—C1	112.04 (15)		
			
O1—C1—C2—C7	177.99 (17)	O3—S1—C7—C6	−61.72 (19)
N1—C1—C2—C7	−1.9 (2)	N1—S1—C7—C6	−176.96 (18)
O1—C1—C2—C3	−2.1 (3)	O1—C1—N1—S1	−174.70 (15)
N1—C1—C2—C3	178.04 (16)	C2—C1—N1—S1	5.15 (19)
C7—C2—C3—C4	0.8 (3)	O1—C1—N1—Ni1	11.1 (3)
C1—C2—C3—C4	−179.07 (16)	C2—C1—N1—Ni1	−169.10 (11)
C2—C3—C4—F1	178.87 (14)	O2—S1—N1—C1	110.32 (13)
C2—C3—C4—C5	0.0 (3)	O3—S1—N1—C1	−122.18 (13)
F1—C4—C5—C6	−179.76 (16)	C7—S1—N1—C1	−5.56 (14)
C3—C4—C5—C6	−0.9 (3)	O2—S1—N1—Ni1	−75.62 (11)
C4—C5—C6—C7	0.9 (3)	O3—S1—N1—Ni1	51.87 (12)
C3—C2—C7—C6	−0.7 (3)	C7—S1—N1—Ni1	168.50 (10)
C1—C2—C7—C6	179.18 (16)	O5^i^—Ni1—N1—C1	−48.82 (14)
C3—C2—C7—S1	178.11 (14)	O5—Ni1—N1—C1	131.18 (14)
C1—C2—C7—S1	−1.98 (18)	O4—Ni1—N1—C1	−137.32 (14)
C5—C6—C7—C2	−0.2 (3)	O4^i^—Ni1—N1—C1	42.68 (14)
C5—C6—C7—S1	−178.74 (14)	O5^i^—Ni1—N1—S1	137.74 (10)
O2—S1—C7—C2	−111.27 (13)	O5—Ni1—N1—S1	−42.26 (10)
O3—S1—C7—C2	119.56 (12)	O4—Ni1—N1—S1	49.24 (10)
N1—S1—C7—C2	4.32 (14)	O4^i^—Ni1—N1—S1	−130.76 (10)
O2—S1—C7—C6	67.46 (19)		

Symmetry codes: (i) −*x*+1, −*y*+1, −*z*+1.

### Hydrogen-bond geometry (Å, °)

**Table:**

*D*—H···*A*	*D*—H	H···*A*	*D*···*A*	*D*—H···*A*
O4—H4A···O2	0.81 (3)	2.51 (3)	3.1436 (19)	137 (3)
O4—H4A···F1^ii^	0.81 (3)	2.54 (3)	3.0910 (19)	127 (3)
O4—H4B···O3^iii^	0.78 (3)	2.17 (3)	2.8985 (18)	158 (3)
O5—H5A···O3	0.80 (3)	2.10 (3)	2.8346 (18)	155 (3)
O5—H5A···F1^iv^	0.80 (3)	2.59 (3)	3.1050 (17)	124 (2)
O5—H5B···O1^i^	0.81 (3)	2.13 (3)	2.793 (2)	139 (2)
O5—H5B···O2^iii^	0.81 (3)	2.44 (3)	2.9541 (18)	122 (2)

Symmetry codes: (ii) −*x*, −*y*, −*z*; (iii) −*x*, −*y*+1, −*z*+1; (iv) −*x*, −*y*+1, −*z*; (i) −*x*+1, −*y*+1, −*z*+1.
